# Adenitis as Initial Mycobacterium marinum Presentation

**DOI:** 10.7759/cureus.41833

**Published:** 2023-07-13

**Authors:** Fernando Oliveira e Silva, Sara Lacerda Pereira, Ana Sofia Santos, António Sarmento, Lurdes Santos

**Affiliations:** 1 Infectious Diseases, Centro Hospitalar Universitário de São João, Porto, PRT; 2 Medicine, Faculdade de Medicina da Universidade do Porto, Porto, PRT; 3 Infectious Diseases, Instituto Português de Oncologia do Porto Francisco Gentil, Porto, PRT; 4 Nephrology and Infectious Diseases R&D, i3S - Instituto de Investigação e Inovação em Saúde, Porto, PRT

**Keywords:** nontuberculous mycobacter, mycobaterium marinum, water-borne disease, adenitis, atypical mycobacteria

## Abstract

*Mycobacterium marinum* is a ubiquitous and opportunist agent that may cause infections related to water activities in humans. It causes mainly skin and soft tissue infections, and other forms of presentation are uncommon. A 27-year-old man presented to the Emergency Department of a tertiary hospital due to a cervical foreign-body sensation that evolved into right cervical swelling and consumption symptoms. He was a waiter on a cruise in the Douro river. Weeks after the initial presentation, the diagnosis of *Mycobacterium marinum* infection was made by positive nucleic acid amplification tests (NAAT) in tissues obtained by excisional biopsy of cervical adenopathy. Treatment with rifampicin and clarithromycin was started. The symptoms improved, and there was a decrease in the adenopathy number and size. Although *Mycobacterium marinum* adenitis as initial presentation of the disease is rare, the identification of the agent by NAAT and favorable response to treatment supported the diagnosis.

## Introduction

*Mycobacterium marinum* is a ubiquitous and opportunist agent that causes infection in freshwater and marine fishes [1, 2]. In humans, it is a possible cause of extrarespiratory infections caused by nontuberculous mycobacteria, and it is highly associated with water activities, namely, swimming in non-chlorinated pools or aquarium handling [2]. It causes mainly skin and soft tissue infections, and other forms of presentation are uncommon [2, 3]. Documenting rare manifestations of this infection and understanding the context in which they arise are important to help other colleagues to establish the diagnosis. Furthermore, there are no well-defined international guidelines regarding the treatment of this disease, so the experience of case reports is crucial to optimize its approach.

## Case presentation

**Figure 1:**
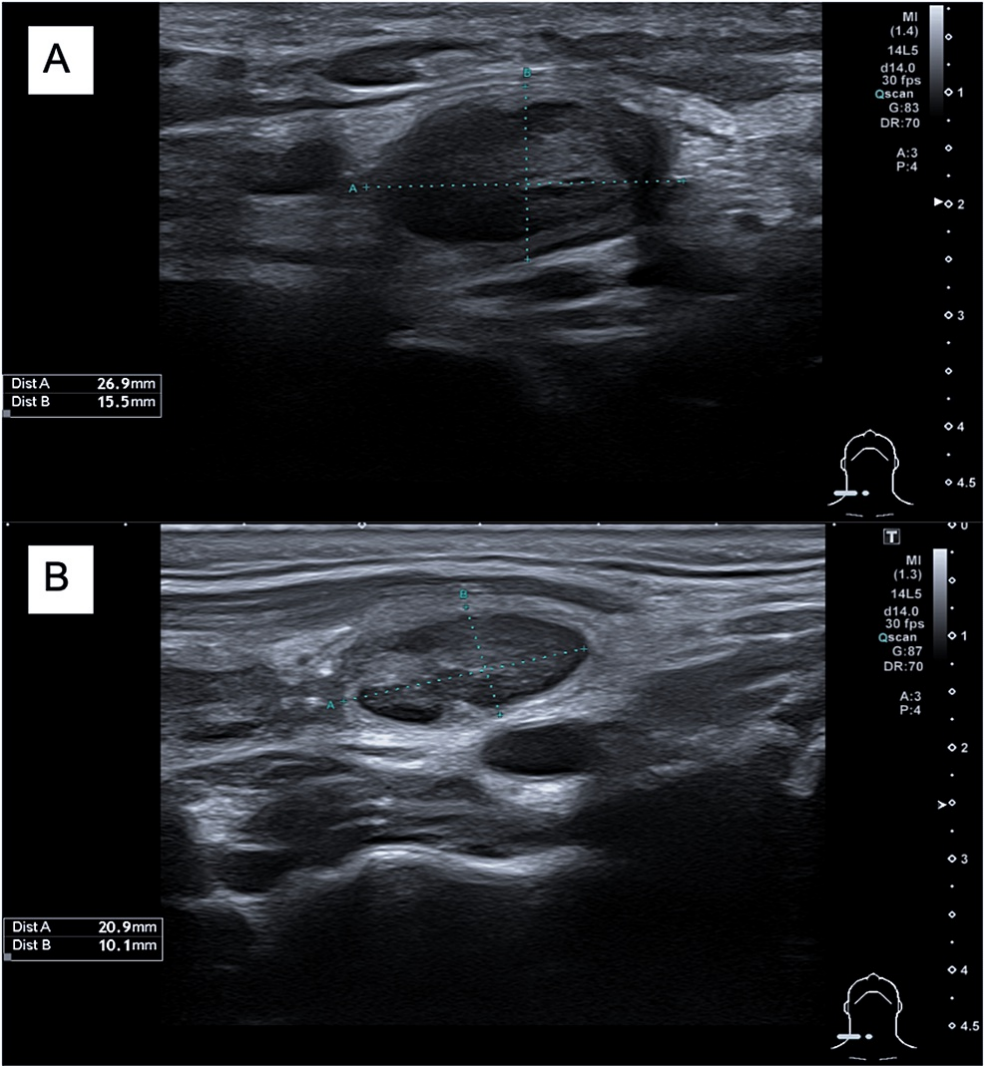
Neck ultrasound Adenopathy in the right lateral cervical region with globose morphology and hypertrophy of the cortical area: the largest ones with 27 x 16 millimeters (A) and 21 x 10 millimeters (B).

A 27-year-old man, previously healthy, presented to the Emergency Department (ED) of a tertiary hospital due to throat discomfort and foreign body sensation. The oropharynx inspection, neck palpation, rhinoscopy, and indirect laryngoscopy made by an otolaryngologist were normal, and the patient was discharged. A few weeks later, the patient noticed a right painless neck swelling and started with asthenia, weight loss, and night sweats. No fever was detected. Six months after, as the symptoms and the neck swelling continued to get worse, he went back to the ED. A neck ultrasound was performed and indicated suspicious cervical adenopathy without associated inflammatory signs (Figure 1).

**Figure 2:**
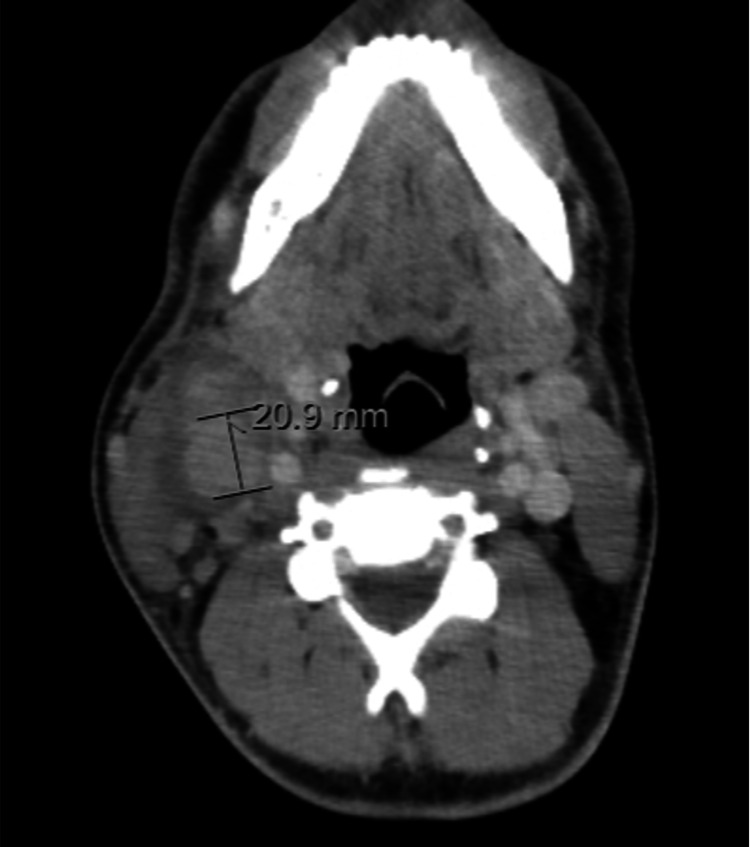
Neck CT scan Cervical adenopathy, mainly globose and atypical right jugular-carotid lymph nodes, the largest one with 21 millimeters.

**Figure 3:**
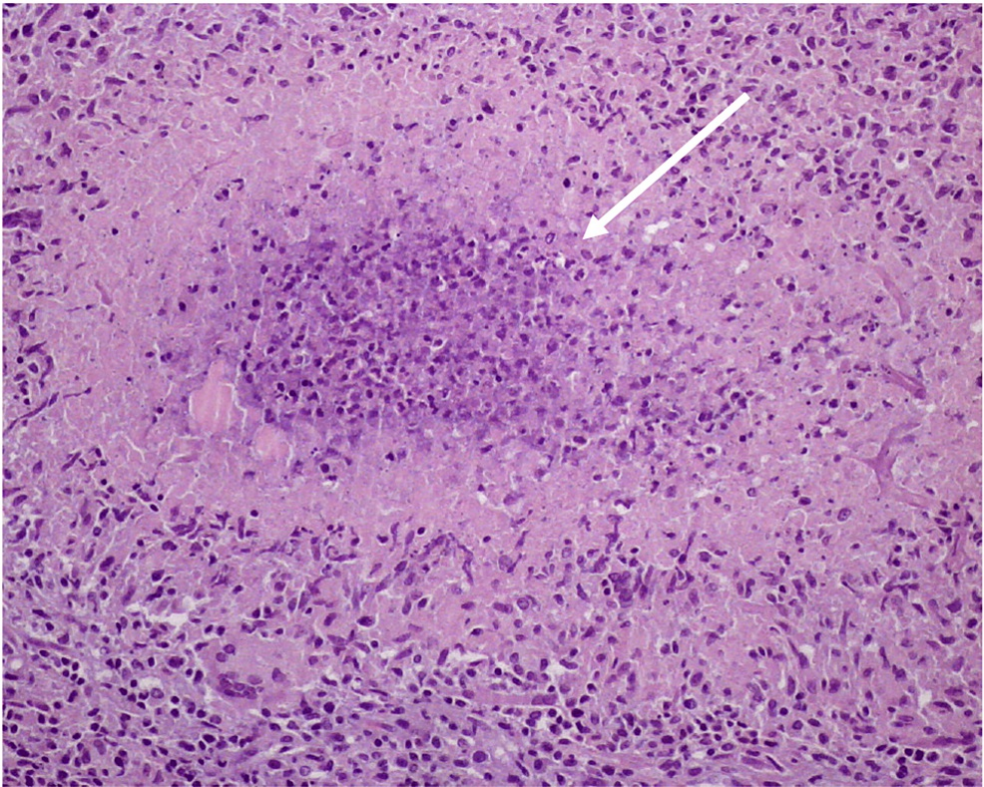
Histologic exam of a cervical lymph node Epithelioid granuloma with central necrosis (arrow). Hematoxylin- and eosin-stained tissue section, 200x magnification.

He was discharged and referred to a hematology consultation for diagnostic research. The study in the outpatient clinic revealed negative human immunodeficiency virus (HIV) screening and neck-chest-abdomen-pelvis computed tomography (CT) without abnormal changes, besides cervical adenopathy (Figure 2) and hepatomegaly. After an inconclusive fine needle aspiration biopsy, an excisional biopsy of a cervical lymph node was performed that showed a necrotizing granulomatous inflammatory lesion, without signs of malignancy, suggestive of an infectious process (Figure 3), with negative microscopic observation with Ziehl-Neelsen, Grocott, and periodic acid-Schiff stains. No samples for culture tests were collected, and the patient was referred to an infectious diseases (ID) consultation. One month later, before the ID appointment, the patient came back to the ED due to intense pain in the anterior side of both legs related to inflammatory nodules. He was hospitalized in the ID ward for a study of erythema nodosum and cervical adenopathy. 

The patient was living in an urban environment, without pets, and the only animals that he contacted were his girlfriend’s cats, very sporadically. He was a waiter on a cruise in the Douro river, where he traveled for several months. The patient also stated that he was responsible for handling the fish and seafood for some of the guest’s meals.

At the time of the hospitalization, he already had lost 8 kilograms (about 9% of weight loss) since the beginning of the symptoms and maintained severe night sweats. Multiple blood tests were performed. Serology for HIV, hepatitis C virus, syphilis, cytomegalovirus, and toxoplasmosis were negative, and he had immunization to hepatitis B virus, Epstein-Barr virus, and varicella-zoster virus. The complete blood count and serum biochemistry were unremarkable, besides an elevation of C-reactive protein (25 mg/L for a normal value inferior to 3 mg/L). The autoimmunity study had no alterations. A new excisional biopsy of a cervical lymph node was performed, and the microbiological study was extended with the identification of *Mycobacterium marinum* by nucleic acid amplification tests (NAAT), namely, GeneXpert® polymerase chain reaction (PCR), despite negative mycobacterial culture tests. GeneXpert® PCR results for *Mycobacterium tuberculosis* were negative.

During the hospitalization, the patient initiated nonsteroidal anti-inflammatory drugs with the improvement of inflammatory nodules on both legs. He was discharged and referred to an ID consultation and to a pulmonology diagnostic center, where mycobacterial infections are treated in Portugal. The patient was treated with a combination of rifampicin 600 mg once daily and clarithromycin 500 mg twice daily for six months, with improvement of his clinical condition, namely, resolution of symptoms and erythema nodosum and a decrease in the adenopathy number and size. After stopping treatment, he maintained surveillance every six months and currently, three years later, he has not had clinical worsening.

## Discussion

Tuberculosis is the main mycobacterial infection in Portugal, and this country has an incidence rate of 14.2 cases per 100,000 inhabitants, the highest rate in Western Europe [4]. In the case of adenopathy accompanied by consumption symptoms, tuberculosis is the main clinical suspicion among infections, and other differential diagnoses may be overlooked and thus not treated properly.

Infection by *Mycobacterium marinum* in European Union countries is uncommon, and this agent accounts for only about 1.3% of the nontuberculous mycobacteria isolates [5]. The disease presents mainly as a skin and soft tissue infection, either as a single papulonodular isolated lesion or as a sporotrichoid manifestation form. The spread to deeper structures is rare, and in about 15% of cases, it may manifest as adenitis related to skin involvement [6]. Our patient manifested an unusual form of the disease, adenitis without skin infective lesions, that could be unnoticed if he did not have a relevant epidemiological context. He also had erythema nodosum, which is considered an immunologic response to multiple conditions, such as tuberculosis or other mycobacterial infections, including *Mycobacterium marinum* [7].

Suspicion of this infection should be raised if the patient has a history of exposure to fishes, water-living animals, or aquaria [2]. In one study, despite several cases were related to skin injuries, most patients just had a history of aquaria exposure [8]. Although, in the past, it was a likely source of infection, currently the disease related to swimming pools is uncommon, probably due to an improvement in water disinfection practices [2]. The patient’s occupation on the ship with the task of handling the fish and seafood was important information that guided the diagnostic study.

The diagnosis of *Mycobacterium marinum* infection is based on a combination of clinical symptoms, exposure history, and laboratory exams. It can be very difficult to obtain if the patient does not present a relevant epidemiological context; as in that case, the index of suspicion is very low [8]. The definite diagnosis requires a positive culture [2], but it is not the only laboratory criterion.

*Mycobacterium marinum* is a slow-growing mycobacterium that belongs to group 1 of the Runyon classification. It may need several weeks to grow on culture, and the optimal temperature is low (28-30ºC) [9], which explains why several cultures are negative, as in the case of our patient. If there is suspicion of infection by this agent, the laboratory should be advised to obtain cultures at two different incubation temperatures (28-30ºC and 35-37ºC) to maximize the growth and identification of the potential etiological agent [9]. Direct microscopic examination after Ziehl-Neelsen staining is rarely positive and cannot differentiate *Mycobacterium marinum* from other mycobacteria [2]. Recently, the use of molecular biology techniques, particularly NAAT, have been applied to identify this agent [10, 11]. Thus, laboratories with this capacity should expand the study to a molecular level to reach the diagnosis more quickly. In the reported case, the use of NAAT was essential to obtain the diagnosis. The histological exam may reveal granulomas, with or without fibrinoid necrosis, that are suggestive, but not pathognomonic, of mycobacterial infection [2]. In the related case, the granulomas observed in the histologic exam misguided thinking toward tuberculosis, which demonstrates the importance of a detailed anamnesis.

Treatment of *Mycobacterium marinum* infections is not standardized. It appears to have a multidrug resistance pattern, particularly to most of the drugs included in the first-line treatment of tuberculosis (isoniazid, ethambutol, and pyrazinamide) [12]. There is no apparent risk of significant mutations that can lead to antibiotic resistance, so routine susceptibility testing is not recommended [9]. Treatment is chosen mainly on personal experience, and although monotherapy may be used in selected cases of superficial skin infections, most authors recommend combination therapy [1-3, 9]. *Mycobacterium marinum* isolates are susceptible to rifampicin, rifabutin, tetracyclines, clarithromycin, moxifloxacin, imipenem, sulfamethoxazole, and amikacin [12]. There is a need for therapeutic trials in humans to show evidence of some of the antibiotics *in vivo*, but a combination of rifampicin and clarithromycin is a recommended regimen in infections extended to deeper structures [2]. The period of treatment should be long, until two months after the resolution of lesions and symptoms [2, 9]. The role of surgery is controversial, but it may be beneficial in deeper infections for source control [13]. Alternative therapies, namely, cryotherapy, X-ray therapy, electrodesiccation, photodynamic therapy, and local hyperthermia, have also been described and recommended in selected cases [3]. The favorable response to the recommended antibiotic treatment, in this case, reinforced the diagnosis.

Prevention is key to diminish the incidence of *Mycobacterium marinum* infections. Swimming pools must have free chlorine levels according to the current recommendations [14]. Individual measures are important with fish and aquarium handling, particularly wearing gloves during these activities [2], which were recommended to our patient since this incident.

## Conclusions

*Mycobacterium marinum* adenitis as the initial presentation of the disease is rare. It may be overlooked if the patient does not have a clear epidemiological context or if the patient’s activities and occupations are not explored. In the presented case, despite culture exams being negative, the agent identification by NAAT and the favorable response to the implemented treatment support the diagnosis. This case highlights the importance of molecular biology methods in the diagnosis of *Mycobacterium marinum* infection, particularly atypical presentations. It also reinforces that the treatment applied in other presentations of the disease, namely, skin manifestations, is also effective in adenitis by this agent.

## References

[1] Hashish E, Merwad A, Elgaml S (2018). Mycobacterium marinum infection in fish and man: Epidemiology, pathophysiology and management; a review. Vet Q.

[2] Aubry A, Mougari F, Reibel F, Cambau E (2017). Mycobacterium marinum. Microbiol Spectr.

[3] Rallis E, Koumantaki-Mathioudaki E (2007). Treatment of Mycobacterium marinum cutaneous infections. Expert Opin Pharmacother.

[4] Direcção Geral de Saúde (2023). Direcção Geral de Saúde - Programa Nacional para a Tuberculose. Relatório de vigilância e monitorização da tuberculose em Portugal - Dados definitivos 2020. definitivos.

[5] van der Werf MJ, Ködmön C, Katalinić-Janković V (2014). Inventory study of non-tuberculous mycobacteria in the European Union. BMC Infect Dis.

[6] Aubry A, Chosidow O, Caumes E, Robert J, Cambau E (2002). Sixty-three cases of Mycobacterium marinum infection: Clinical features, treatment, and antibiotic susceptibility of causative isolates. Arch Intern Med.

[7] Schwartz RA, Nervi SJ (2007). Erythema nodosum: A sign of systemic disease. Am Fam Physician.

[8] Jernigan JA, Farr BM (2000). Incubation period and sources of exposure for cutaneous Mycobacterium marinum infection: Case report and review of the literature. Clin Infect Dis.

[9] Griffith DE, Aksamit T, Brown-Elliott BA (2007). An official ATS/IDSA statement: Diagnosis, treatment, and prevention of nontuberculous mycobacterial diseases. Am J Respir Crit Care Med.

[10] Tortoli E, Nanetti A, Piersimoni C (2001). Performance assessment of new multiplex probe assay for identification of mycobacteria. J Clin Microbiol.

[11] Russo C, Tortoli E, Menichella D (2006). Evaluation of the new genotype Mycobacterium assay for identification of mycobacterial species. J Clin Microbiol.

[12] Aubry A, Jarlier V, Escolano S, Truffot-Pernot C, Cambau E (2000). Antibiotic susceptibility pattern of Mycobacterium marinum. Antimicrob Agents Chemother.

[13] Bhatty MA, Turner DP, Chamberlain ST (2000). Mycobacterium marinum hand infection: Case reports and review of literature. Br J Plast Surg.

[14] (2023). Centers for Disease Control and Prevention. Water treatment and testing. https://www.cdc.gov/healthywater/swimming/residential/disinfection-testing.html.

